# A guideline for the inpatient care of children with pyelonephritis

**DOI:** 10.4103/0256-4947.68549

**Published:** 2010

**Authors:** Aftab S. Chishti, Erich C. Maul, Rubén J. Nazario, Jeffrey S. Bennett, Stefan G. Kiessling

**Affiliations:** a From the Department of Pediatrics, Division of Nephrology, University of Kentucky, Lexington, Kentucky, USA; b From the Section of Inpatient Pediatrics, Division of General Academic Pediatrics, University of Kentucky, Lexington, Kentucky, USA; c From the Division of Infectious Disease, University of Kentucky, Lexington, Kentucky, USA

## Abstract

**BACKGROUND AND OBJECTIVES:**

Febrile urinary tract infections and pyelonephritis are common in children and frequently lead to hospitalization for management, especially in the child who appears toxic. The American Academy of Pediatrics (AAP) practice parameter on the diagnosis, treatment and evaluation of the initial urinary tract infection in febrile infants and young children provides experience and evidence-based guidelines for the practitioner caring for children between the ages of 2 months to 2 years. No established guideline exists for older children and the AAP guideline does not specifically focus on inpatient care.

**METHODS:**

We conducted a comprehensive review of recently published literature and practice guidelines to develop a consensus on the inpatient diagnosis and management of children with pyelonephritis.

**RESULTS:**

Eight recommendations are proposed for the diagnosis and management, including revised guidelines for the imaging studies postpyelonephritis on the basis of current best evidence.

**CONCLUSION:**

Proper diagnosis of pyelonephritis, timely initiation of appropriate therapy and identification of children at risk for renal injury will help to reduce immediate as well as long-term complications due to chronic kidney disease.

Urinary tract infections (UTIs) in children commonly present with fever and are often associated with anatomic or functional abnormalities of the urinary system. The high risk of pyelonephritis in the presence of a febrile UTI and possible complications including renal scarring, hypertension, and chronic renal disease, warrants accurate and timely diagnosis, evaluation and therapy. Even though the risk for febrile UTIs and related renal scarring is highest in the first year of life, older children can also develop pyelonephritis and associated long-term renal injury.^1^ Fever, urinary symptoms, flank pain and a suspicious urinalysis should always raise the question of possible pyelonephritis in any age group. UTIs account for 0.7% of office visits and 5% to 14% emergency department visits by children annually.^2^ The overall prevalence of UTI in older children with or without fever is reported to be 7.8%; for febrile females <3 months of age the prevalence is 7.5% and for febrile circumcised males <3 months it is 2.4%, which increases to 20.1% in similar age uncircumcised males. Caucasian infants are more likely to have UTI.^3^

Data from the 2005 report of the North American Pediatric Renal Transplant Collaborative Studies (NAPRTCS) confirm that renal injury from reflux nephropathy and chronic pyelonephritis continues to be a major concern and the most common etiologic factor of potentially preventable long-term renal disease in children.^4^ More than 8% of children enrolled in NAPRTCS with chronic renal disease carried the diagnosis of reflux nephropathy. Many of those need renal replacement therapy and renal transplantation at some point in the future.^4^

It is often difficult to prevent the initial episode of a febrile UTI, because the risk factors are not always overt. Therefore, focus needs to be on prevention of re-infection. About 10% to 15% of children with one febrile urinary tract infection and pyelonephritis will develop renal scarring.^1^ This risk is significantly increased in the presence of recurrent UTI and associated nephro-urologic abnormalities, such as vesicoureteral reflux (VUR). The American Academy of Pediatrics (AAP) has issued a practice parameter on this topic for children between 2 months to 2 years of age,^5^ but no evidence-based guideline exists for the inpatient care of children admitted with a working diagnosis of pyelonephritis. As a significant percentage of children require admission to an inpatient pediatric unit., this article will focus primarily on the care of the hospitalized child.

## Etiology, Pathogenesis and Risk Factors

*Escherichia coli* is the pathogen identified in 80% to 90% of children with a first urinary tract infection and is also the pathogen in two thirds of recurrent UTIs. Other bacterial pathogens include Enterobacteriaceae like *Proteus, Klebsiella*, as well as *Staphylococcus* and *Streptococcus* species (Table 1).^6^–^10^ Most urinary tract infections and consecutive pyelonephritis occur by bacteria ascending retrograde via the perineal-urethral-bladder route and further ascending into the upper urinary tract.^11^ The mechanism of ascent into the upper urinary tract has not been identified so far. To develop pyelonephritis, a combination of two factors needs to occur: colonization of the urinary tract with a potential pathogen, and ascent of the pathogen into the kidney

**Table 1:** Etiologic agents of pediatric urinary tract infections.

Gram-negative organisms	Features
*Escherichia coli*	Most common organism. Causative agent in >80% of first UTIs.
*Klebsiella* species	Second most common organism. Seen more in young infants. Sixteen percent of bacteremic children have underlying urinary tract anomalies.
*Proteus* species	May be more common in males. Three of nosocomial UTIs.
*Enterobacter* species	Cause <2% of UTIs. Mostly nosocomial.
*Pseudomonas* species	Cause <2% of UTIs. Most prevalent non-enteric gram-negative pathogen.
**Gram-positive organisms**	
*Enterococus* species	Uncommon >30 days of age. Commonest gram-positive pathogen. Up to 5% of UTIs.
Coagulase-negative *Staphylocccus*	Uncommon in childhood. If suspicion is high for UTI, adjust antibiotic therapy to cover; otherwise, a repeat culture is prudent.
*Equivalent Staphlococcus saprophyticus*	Rare prior to puberty. Up to 15% of adolescent female UTI.
*Staphylococcus aureus*	Uncommon >30 days of age.
Group B streptococci	Uncommon in childhood.

Several risk factors for UTIs have been identified and discussed in the past. These include younger age, gender, fecal and perineal colonization, sexual activity, anatomic abnormalities, functional abnormalities, and immune system suppression.^12^ Non-anatomic risk factors, such as constipation, dysfunctional voiding syndrome, and exposure to potential bladder irritants, may play significant roles in the pathogenesis of illness for individual children. Nevertheless, it needs to be pointed out that these factors only increase the risk for UTI, and therefore secondary mechanisms need to be present for bacteria to ascend into the upper urinary tract, causing clinical pyelonephritis.

After the first febrile UTI, 30% to 50% of all children are diagnosed with VUR. With routine ultrasound evaluation starting in pregnancy, only 1% or less of individuals are found to have anatomic obstruction as the etiologic factor.^13^ It appears that children at highest risk often have abnormalities in both mechanisms that usually work in combination to protect individuals from infection: a properly functioning antegrade urine washout and low bacterial adherence to the urinary tract. Disruption of this mechanism will increase the chances for bacteria to ascend into the bladder or the upper urinary tract.

## Recommendations: Clinical Findings in Children with Acute Pyelonephritis

*Recommendation 1:* As clinical findings of pyelonephritis can be very nonspecific, a urinalysis and urine culture should be part of the evaluation of any child admitted to the inpatient hospital with fever of unclear source, even in the absence of classic symptoms of pyelonephritis. In children less than 2 years of age, and especially neonates, signs of systemic infection including decrease in activity, poor appetite, lethargy, diarrhea and hypotension, are far more common than in older children.^5^ Occasionally asymptomatic jaundice is the only presenting sign in neonates. Between the ages of 2 years and 5 years, the presenting complaints can be nonspecific, with complete absence of urinary symptoms, with fever and abdominal pain as the only presenting complaints. Most children older than the age of 5 years present with classic findings suggestive of pyelonephritis. These usually are dysuria, urinary frequency and urgency, fever, flank pain and possibly hematuria.^12^ Although fever and flank pain are commonly seen in patients with pyelonephritis, these findings are neither sensitive nor specific. As many as 25% of children who have no classic signs or symptoms are ultimately confirmed to have upper urinary tract disease, and up to 50% of patients with flank pain have no evidence of pyelonephritis. Since it is often difficult to have specific signs and symptoms of UTI, a high index of suspicion is required to make the correct diagnosis.

## Recommendations: Diagnosis

*Recommendation 2:* A midstream clean catch in toilet-trained children or a catheterized urine sample in patients unable to reliably collect a clean catch specimen should be sent for urinalysis and culture before initiation of antibiotic therapy. A urine sample should be collected and sent for culture before initiation of empiric antibiotic therapy;^12^ only one dose of antibiotic could potentially lead to sterilization of the urine and cloud the proper diagnosis. The urine sample can be collected by a variety of methods depending on the age and toilet training status of the child. If a midstream clean catch specimen in a toilet-trained child is not possible, a catheterized specimen should be obtained. Bag specimens may be considered in children more than 3 months of age, especially if the index of suspicion is low and use of empiric antibiotics are not being considered. In one study, the sensitivities of bag urinalysis were found to be higher than catheterized specimen (85% vs. 71% with a *P* value of .003) even though the specificity was definitely lower (62% vs. 97% with a *P* value of <.001) in children <2 years of age.^14^ Supra-pubic aspiration of the bladder, which is performed less frequently in clinical practice these days, remains a valid option up to 18 to 24 months, as thereafter the bladder becomes a completely pelvic organ.^15^ It can easily be done with minimal risks, but the success rate in obtaining an adequate amount of urine is rather limited (40%), so its use has gradually fallen out of favor.

Four major determinants in the urinalysis are supportive of the diagnosis of a UTI: (1) a positive urinary leukocyte esterase (revealing the presence of white blood cells in the urine), which has a sensitivity and specificity of about 75%; (2) a positive urinary nitrite (dietary nitrates are reduced to nitrite by many gram-negative urinary bacteria) has a sensitivity 60% although the specificity is 100%; (3) more than 5 white blood cells per high-powered field on microscopic examination of the spun urinary sediment; and (4) any bacteria seen on a high-powered examination of the spun urinary sediment has a sensitivity of 93% and specificity of 95% (Table 2).^16^,^17^

**Table 2:** Predictive value of urinalysis components.

Tests	Sensitivity % (range)	Specificity % (range)
Nitrites	50 (16-72)	98 (95-100)
Leukocyte esterase	83 (64-89)	84 (71-95)
>5 WBC/HPF	67 (55-87)	79 (77-84)
Any organism on Gram stain	93 (80-98)	95 (87-100)

Urine culture remains the gold standard for diagnosing UTI; a positive urine culture requires the growth of a single organism in a significant quantity. On a clean catch specimen, more than 10 000 colonies in boys suggest likely infection and 100 000 colonies in girls makes the diagnosis of an infection likely, whereas greater than 10 000 colonies on a catheterized specimen makes the diagnosis of an infection very likely.^5^ It needs to be emphasized that lower colony counts (>1000) can also be significant under clinical circumstances and do not rule out a possible infection.

## Recommendations: Inpatient Therapy of Pyelonephritis

*Recommendation 3:* After collection of urine for culture, antibiotic therapy should be initiated without delay if the urinalysis is suspicious and/or clinical findings are consistent with a febrile upper urinary tract infection. Prompt initiation of empiric broad-spectrum systemic antibiotic therapy to achieve sufficient antimicrobial drug levels has been shown to reduce the risk of complications, especially renal scarring.^18^,^19^ Once the kidney is involved in the febrile UTI, timing of antibiotics does not seem to reduce the risk for renal scarring, so that the overall goal needs to be to prevent renal infection.^20^ Toxic-appearing children (high fever, flank pain) should therefore be admitted to the inpatient hospital for initiation of intravenous antibiotic therapy. Other indications for hospital admission include the inability to tolerate oral therapy and the possibility of medication non-compliance. Children with a history of underlying urinary tract pathology including renal dysfunction should also be admitted for inpatient intravenous therapy. Children older than 2 months with pyelonephritis who are non-toxic may receive effective therapy by the oral route, and may not require admission if there is confidence in the compliance of the family and reliability of outpatient follow-up.^21^ Most admitted children can be successfully treated with a short course of intravenous antibiotics^22^ until the urine culture results are available. Intravenous antibiotic therapy is needed for at least 48 to 72 hours or until the child is clinically improving and afebrile for more than 24 hours; then transition to oral antibiotics therapy is considered. Routine follow-up urine cultures are not recommended, as most urine cultures are sterile (negative) after 24 hours of antibiotic therapy.^13^ However, it may be useful to re-culture the urine in children who are clinically not improving.

Tables 3a and 3b are a list of commonly used antibiotic agents in children with pyelonephritis. Usually a combination of ampicillin and gentamicin is the standard empiric therapy. The risk of aminoglycoside-induced nephrotoxicity in the setting of pyelonephritis has been reported in an animal model;^23^ therefore, we recommend using aminoglycosides with caution in patients demonstrating any evidence of renal dysfunction. Ampicillin combined with cefotaxime or ceftriaxone are appropriate alternative empiric regimens. To ensure proper dosing of antibiotics cleared by the kidneys, the glomelular filtration rate (GFR) is estimated according to the Schwartz formula before therapy is initiated.

**Table 3a:** Parenteral antibiotic therapy options for acute pyelonepphritis.

Antimoicrobal agent	Dose (mg/kg/day)	Frequency (hourly)
**Penicillins**		
Ampicillin^a^	100-200	Q6
Ticarcillin	50-200	Q4-8
**Aminoglycosides**		
Gentamicin	7.5	Q8
Amikacin	22.5	Q8
**Cephalosporins**		
Cefazolin	50-100	Q6
Cefotaxime	100-200	Q8
Ceftriaxone^a^	50-75	Q12-24
Ceftazidime	90-150	Q8-12
Cefipime	100	Q12
**Fluoroquinolones**		
Ciprofloxacillin	18-30	Q8

a No dose adjustment in azotemia.

**Table 3b:** Oral antibiotic therapy options for acute pyelonephritis.

Antimoicrobal agent	Dose (mg/kg/day)	Frequency (hourly)
**Penicillins**		
Amoxicillin^a^	20-40	Q8
Augmentin^a^	20-40	Q8
**Cephalosporins**		
Cephalexin	25-100	Q6-8
Cefadroxil	30	Q12
Cefuroxime	75-150	Q12
Cefixime	16 on Day 1 then 8	Q12 on Day 1 then Q24
**Sulphonamides**		
Trimethprim-Sulphamethoxazole	6-12 Based on trimethoprim	Q12
**Fluorquinalones**		
Ciprofloxacillin	20-40	Q12
**Others**		
Nitrofurantoin^a^	5-7	Q6

a No dose adjustment in azotemia.

Patients who require bladder catheterization or who have a history of UTI with resistant pathogens may require broader empiric therapy to include *Pseudomonas* coverage. In this context, empiric use of cefipime in lieu of third-generation cephalosporins is appropriate. Ciprofloxacin has been recently approved for treatment of children older than 1 year and it can be considered for use if there is no other option or the child has underlying urologic anomalies.^24^ Empiric oral therapy for a non-toxic patient with pyelonephritis is an option based on available evidence.^25^ Oral cefixime is one of the third-generation cephalosporins currently recommended for this purpose; in addition, cefuroxime or cefdinir can also be used. Cephalexin, while commonly used for UTI in children as empiric therapy, has not been studied prospectively for equivalence with intravenous therapy. It is appropriate, however, for uncomplicated cystitis. Similarly trimethoprim-sulfamethoxazole has been used extensively for UTI therapy, but only 77% of our *E coli* isolates were susceptible. Nitrofurantoin has unreliable kidney tissue penetration, and should not be used therapeutically for pyelonephritis.

Antibiotic treatment, whether intravenous or oral, should immediately be tailored to the sensitivities of the identified pathogen when available. If there is no growth on a properly obtained urine culture (without prior treatment with antibiotics), discontinuation of antibiotics is appropriate and recommended.^21^ Duration of therapy is based on the severity of the disease and how rapidly the patient responds to the initial therapy. In uncomplicated pyelonephritis, shorter courses have been used;^26^ however, it is typically recommended for a minimum of 10 days.

## Recommendations: Supportive Therapy

*Recommendation 4:* Administration of intravenous fluids should be considered in any child admitted for inpatient care with suspected pyelonephritis. Antibiotics are the mainstay of therapy for febrile UTIs. Children with pyelonephritis often appear quite ill with decreased appetite, vomiting and dehydration, which can be worsened by an increase in fluid and caloric needs in the presence of fever. In addition, previous studies have shown that pyelonephritis can be associated with polyuria and a decreased renal concentrating ability, increasing the risk for dehydration.^27^,^28^ Administration of intravenous fluids helps optimize renal perfusion and urine output, which appears to help clearing of the bacteria from the urinary tract. The lack of consensus regarding the optimal intravenous solution is beyond the scope of this review. However, the clinician should evaluate the degree of dehydration and replenish it accordingly. Additional fluids should be estimated based on insensible losses such as fever, increased metabolic rate, and tachypnea. Once the child tolerates oral fluids, intravenous fluids should be discontinued. In our experience, normal saline is optimal to provide bolus intravenous fluids in cases where intravascular volume needs to be restored rather quickly, whereas 5% dextrose and normal or half normal saline are used for maintenance and longer rehydration. To avoid iatrogenic hyponatremia, any solution with less than half normal saline should be used with caution and under controlled clinical circumstances.

*Recommendation 5:* Antipyretics should be used with caution given their potential for additional nephrotoxicity. The fever curve can be a helpful marker for therapy response. Management of fever remains somewhat controversial. Sarrell et al^29^ report that alternating antipyretics is more effective than monotherapy and reduces temperatures faster. Other reports suggest that alternating acetaminophen and ibuprofen can lead to acute and chronic overdoses of antipyretics.^30^ Renal side effects of non-steroidal anti-inflammatory drugs are well known and have been reported in the past.^31^ Non-steroidal medications exert their antipyretic propensity by decreasing the synthesis of prostaglandins, which are important in the regulation of renal bloodflow. A decrease in prostaglandin synthesis could potentially decrease renal blood flow and therefore induce a “pre-renal” physiology, increasing risk of tubular damage. Even though both ibuprofen as well as acetaminophen can alter renal hemodynamics by inhibiting synthesis of prostaglandins and counteracting their renal vasodilator effects, ibuprofen has a more significant effect on glomerular filtration rate than acetaminophen. This could be of importance in inducing additional renal injury, especially in case of sodium and volume depleted state that often accompanies pyelonephritis.^32^ Caregivers need to be reassured that fever is not a disease and that we can use it as a clinical indicator of disease progression.

## Recommendations: Diagnostic Imaging

*Recommendation 6:* Diagnostic imaging after documentation of a febrile UTI by renal ultrasound, dimercaptosuccinic acid (DMSA) scan and/or voiding cystourethrogram (VCUG) should to be individualized depending on presentation, age and gender of the child as well as results of prenatal ultrasonography. The goal of imaging of the urinary tract after a febrile UTI is to identify children at risk for recurrent infection and chronic renal injury. Three standard diagnostic tests are commonly used in clinical practice: renal and bladder ultrasound (RUS), VCUG and renal DMSA scan. The RUS continues to be the most commonly utilized imaging study in the evaluation of a febrile UTI. As outlined by Montini et al,^33^ imaging studies should be used in case the results alter the clinical course of action and improve outcome. A bilateral RUS including complete assessment of the bladder by pre- and post-voiding images has previously been suggested as standard part of the evaluation of febrile UTIs,^34^ but its yield has been questioned in the recent past.^35^–^37^ Diagnostic evaluation by ultrasound may not be needed after a UTI in children who had a prenatal ultrasound confirming the presence of two anatomically normal kidneys.^33^ In a study by Giorgi et al, routine renal ultrasound only altered the management in 4.4% of the patients.^37^ Recently published guidelines by the National Institute of Clinical Excellence (NICE) in the United Kingdom recommend performing a renal ultrasound only in an acute UTI with atypical presentation (defined as UTI with septicemia, poor urine flow, abdominal or bladder mass, elevated serum creatinine level, non *E coli* UTI or UTI that fails to improve with 48 hours of intravenous antibiotics); otherwise it can be deferred or omitted.^39^

Though the overall yield of pathology with RUS might be low and results not predictive of future renal scarring, they can be useful and lead to detection of significant pathology, especially if no prenatal ultrasound was performed.^33^ Renal ultrasound is a non-invasive diagnostic test that can detect significant pathology, including hydronephrosis, acquired urinary tract obstruction and bladder anomalies. Bladder wall thickness, stool pushing against the bladder, and detection of post-void urine residuals can help identify children with dysfunctional elimination, who may be at risk for future urinary tract infections, and may guide the management after the acute illness has resolved.

VCUG remains the diagnostic test of choice for the detection of VUR and its use is widespread in clinical practice. VUR is detected in 25% to 40% of children presenting with a first episode of pyelonephritis.^24^ Multiple studies have shown no significant difference in the rate of detection of VUR with a VCUG performed early (within one week) or late (2-3 weeks) after an episode of acute pyelonephritis.^40^,^41^ The recommendation to perform routine VCUG after febrile UTIs was based on studies linking the presence of reflux to the risk for pyelonephritis and related renal scarring.^42^,^43^ The role of VUR in progression of renal injury leading to scarring may not be as clear as once thought. Data suggest that low grade reflux (up to Grade III) may neither be an indication to treat medically nor surgically.^44^ As a rather invasive test, the usefulness of a routine VCUG needs to be re-examined, especially if reflux by itself is not a risk factor for chronic renal injury. Also, recent data supports the utility of early DMSA scans in the diagnosis of pyelonephritis. Tseng et al have shown in a 10-year retrospective review that only a small number of children with a normal DMSA scan performed within 2 days of the diagnosis of pylonephritis do have reflux, and no child in this study had high grade reflux grade (III-V).^45^

**Figure 1:**
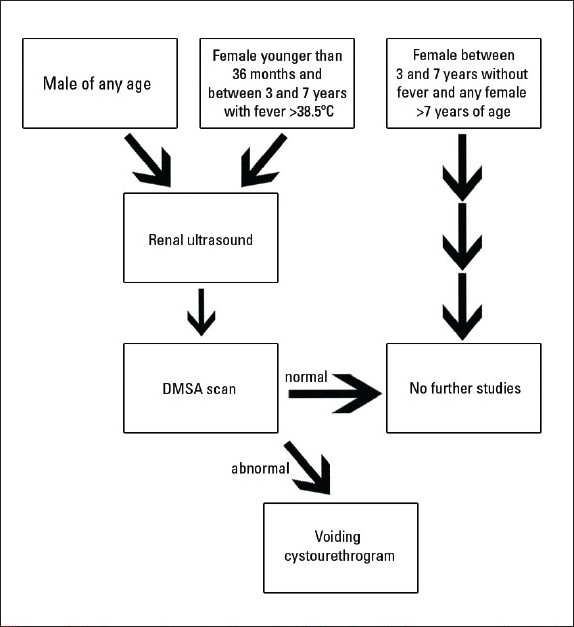
Diagnostic imaging algorithm in children after the first pyelonephritis.

Once a febrile UTI is diagnosed, the question arises whether it has affected the kidneys. A renal DMSA scan, if performed early on, can help support the diagnosis of acute pyelonephritis^46^ or, if delayed for 4-6 months, can detect parenchymal damage sustained by the kidney in the form of renal scarring. DMSA scans have been shown to be several times more sensitive in detecting renal scars than intravenous pyelogram or ultrasound.^47^ As mentioned, DMSA scans might also be of use to decrease the number of more invasive VCUG studies. In a prospective study, Preda et al showed that almost all infants with pyelonephritis and high-grade reflux had an abnormal DMSA scan, while most of the infants who had low-grade reflux on VCUG had normal DMSA scans, suggesting that VCUG should not be routinely used as a screening tool.^47^ Similarly, the NICE guidelines also recommend performing a DMSA scan in all children less than 3 years of age with atypical or recurrent UTI. We propose to reserve the VCUG for children who have abnormal DMSA scans in line with the above evidence, but still find use in performing renal sonograms as it is simple, affordable and noninvasive for all children. A suggested diagnostic algorithm is presented in Figure 1.

## Recommendations: Role of Prophylactic Antibiotics

A very important aspect in the management of children with febrile UTIs are available prevention strategies, including the role of prophylactic antibiotics, correction of dysfunctional elimination and the role of cranberry juice and probiotics.

*Recommendation 7:* Use of prophylactic antibodies remains controversial, especially in the presence of low-grade vesicoureteral reflux. The efficacy of prophylactic antibiotics in preventing recurrent febrile UTIs and pyelonephritis, with the goal to decrease the incidence of chronic renal disease due to scarring with and without VUR, has been discussed widely. Publications in the 1980s supported the use of once a day low dose use of antibiotics for prevention of UTIs.^49^–^51^ Despite their current widespread use, a growing body of evidence in larger, multicenter studies does not support benefit of prophylactic antibiotics in the absence or presence of low-grade VUR.^52^,^53^ Also, a recent systematic review of randomized controlled trials related to the topic found a lack of evidence of a positive benefit for children at risk for developing UTIs.^54^ More importantly, it has been shown that the prolonged use of the prophylactic antibiotics can result in resistant and difficult-to-treat future infections.^55^ Wheeler et al showed in their meta-analysis that the risk of recurrence of UTIs by 1, 2, and 5 years was not significantly different in antibiotic prophylaxis and surgically treated groups, although the combined treatment resulted in a minor reduction of febrile UTI by year 5, but no concomitant reduction in new or progressive renal damage was noted.^56^ Despite certain limitations, results of a recent randomized clinical trial from Italy support the idea that prophylactic antibiotics might not be effective in reducing the risk of recurrent pyelonephritis and incidence of renal scarring in children less than 30 months of age who have grade II to grade IV vesicoureteric reflux.^57^ In addition, the current NICE guidelines^38^ do not advocate the use of prophylactic antibiotics in children with UTIs. In summary, based on all the available evidence, the decision to use prophylactic antibiotics should be discussed and tailored in an individualized fashion; children at high risk for recurrence of UTI including high-grade VUR or severe voiding dysfunction might benefit from prophylactic antibiotics. It is imperative to adequately treat any UTI and aggressively work on any functional risk factors such as constipation or dysfunctional voiding to decrease risk for UTI recurrence.

## Identification and Importance of Functional Abnormalities

Once structural anatomic pathology has been ruled out, focus needs to be on the identification of other risk factors for UTIs and pyelonephritis. The role of constipation in the pathogenesis of febrile UTIs continues to be significant and well documented. Loening-Baucke,^58^ like many others, has shown that aggressive treatment of functional constipation and encopresis in the absence of anatomic abnormalities can lead to complete resolution of urinary tract infections in children. Urinary stasis and incomplete bladder emptying are seen in children who have a tendency to withhold urine and/or have suboptimal hydration. We recommend initiating the evaluation process already in hospitalized children, but certainly most of the management can and will be done during scheduled outpatient follow-up visits.

*Recommendation 8:* There is no clear evidence to either support or reject the use of alternative therapies such as cranberry juice and probiotics. Physicians are often questioned about the use of cranberry juice and/or its extracts in the prevention of UTI. Much of the original research into the mechanism of cranberry’s mechanism of action was focused on the acidification of urine and hippuric acid excretion.^59^,^60^ While much of this original research has been refuted, what is being discovered is that cranberry contains moieties which inhibit or prevent bacterial adhesion to uroepithelium, a crucial first step in the development of any UTI. Liu et al documented the molecular anti-adhesive properties of cranberry juice on certain strains of P-fimbriated *E coli*.^59^ In 1989, Zafriri et al were the first to isolate two compounds responsible for the antiadhesive properties of cranberry. One entity was fructose, which inhibits mannose sensitive adhesions and a second, high molecular weight compound which inhibits the mannose resistant adhesions; this was termed proanthocyanidin.^61^ However, there is no current pediatric literature to support or refute the use of cranberry products for preventing UTIs. Two studies have suggested a lack of efficacy in children with neurogenic bladder, but no prospective trials have evaluated patients with recurrent UTIs.^62^,^63^ Further research in this area would be of benefit.

## Summary

Febrile UTI and pyelonephritis are among the most common potentially serious bacterial infections in children. Nephronogenesis is usually complete at 36 weeks of gestation and cannot be resumed; therefore, significant renal injury secondary to infection or other causes can lead to irreversible renoparenchymal injury with progressive loss of kidney function over time so efforts should focus on prevention. Especially in very sick children, the timely diagnosis and optimal management of pyelonephritis can decrease the risk of long-term renal injury. In addition to timely diagnosis and appropriate medical therapy, prevention of future infections by detecting structural anatomic or functional abnormalities is crucial. Minimizing the risk for long-term renal disease by using as little invasive testing as possible is certainly one of the major goals. The past approach of detection and follow-up on structural anomalies using radiological investigations has more recently come under immense scrutiny as solid evidence of benefit is uniformly lacking and to date most recommendations are based on limited data. This will hopefully change as more evidence is gathered. This guideline might help by initiating larger, prospective studies.
